# Transgenic inhibition of interleukin-6 *trans*-signaling does not prevent skeletal pathologies in mucolipidosis type II mice

**DOI:** 10.1038/s41598-021-82802-3

**Published:** 2021-02-11

**Authors:** Lena Marie Westermann, Anke Baranowsky, Giorgia Di Lorenzo, Tatyana Danyukova, Jamie Soul, Jean-Marc Schwartz, Gretl Hendrickx, Michael Amling, Stefan Rose-John, Christoph Garbers, Thorsten Schinke, Sandra Pohl

**Affiliations:** 1 Department of Osteology and Biomechanics, University Medical Center Hamburg-Eppendorf, 20246 Hamburg, Germany; 2 Clinic of Trauma and Orthopedic Surgery, University Medical Center Hamburg-Eppendorf, 20246 Hamburg, Germany; 3 Skeletal Research Group, Biosciences Institute, Newcastle University, Newcastle upon Tyne, NE1 7RU UK; 4 School of Biological Sciences, Faculty of Biology, Medicine and Health, University of Manchester, Manchester, M13 9PL UK; 5 Institute of Biochemistry, Christian-Albrechts-University of Kiel, 24098 Kiel, Germany; 6 Department of Pathology, Otto-Von-Guericke-University Magdeburg, 39120 Magdeburg, Germany; 7 Present Address: Telethon Institute of Genetics and Medicine (TIGEM), 80078 Pozzuoli, Italy; 8 Present Address: Center of Medical Genetics, Antwerp University Hospital and University of Antwerp, 2610 Edegem, Belgium

**Keywords:** Cell biology, Molecular biology, Diseases, Molecular medicine

## Abstract

Severe skeletal alterations are common symptoms in patients with mucolipidosis type II (MLII), a rare lysosomal storage disorder of childhood. We have previously reported that progressive bone loss in a mouse model for MLII is caused by an increased number of bone-resorbing osteoclasts, which is accompanied by elevated expression of the cytokine interleukin-6 (IL-6) in the bone microenvironment. In the present study we addressed the question, if pharmacological blockade of IL-6 can prevent the low bone mass phenotype of MLII mice. Since the cellular IL-6 response can be mediated by either the membrane-bound (classic signaling) or the soluble IL-6 receptor (*trans*-signaling), we first performed cell culture assays and found that both pathways can increase osteoclastogenesis. We then crossed MLII mice with transgenic mice expressing the recombinant soluble fusion protein sgp130Fc, which represents a natural inhibitor of IL-6 *trans*-signaling. By undecalcified histology and bone-specific histomorphometry we found that high circulating sgp130Fc levels do not affect skeletal growth or remodeling in wild-type mice. Most importantly, blockade of IL-6 *trans*-signaling did neither reduce osteoclastogenesis, nor increase bone mass in MLII mice. Therefore, our data clearly demonstrate that the bone phenotype of MLII mice cannot be corrected by blocking the IL-6 *trans*-signaling.

## Introduction

The maintenance of healthy skeletal structure and function during development, growth and adulthood is achieved by resorption of aged bone by osteoclasts and formation of new bone by osteoblasts. Tightly regulated bone turnover depends on the balance of these two cellular systems. Osteoclasts are highly specialized multinucleated cells derived from hematopoietic precursor cells, whereas osteoblasts are mononuclear cells that arise from a mesenchymal stem cell lineage^1,2^. Besides, a subset of osteoblasts undergoes terminal differentiation into osteocytes, which form a cellular network within the mineralized bone matrix and regulate bone remodeling and mineral homeostasis^3^. The differentiation of pre-osteoclastic cells to mature osteoclasts requires stimulating factors synthesized and secreted by osteoblasts. Among them, receptor activator of nuclear factor-κB ligand (RANKL) was found to be the key stimulant of canonical osteoclastogenesis^4,5^. RANKL binds to its receptor RANK, which is expressed on the surface of osteoclast precursors, and subsequently activates a variety of downstream signaling pathways required for the formation and maturation of osteoclasts^6^. However, a number of other cytokines have been identified to induce non-canonical osteoclastogenesis with IL-6 being one of them^7–10^. Indeed, increased production and/or action of IL-6 have been implicated in the pathogenesis of disease states characterized by excessive bone resorption in adulthood such as postmenopausal osteoporosis and Paget’s disease^11–13^.

Depending on the cell type, IL-6 signal transduction occurs via classic and *trans*-signaling^14^. In the classic signaling pathway, IL-6 binds to the membrane-bound IL-6 receptor (IL-6R) and subsequently associates with ubiquitously expressed membrane-bound, homodimeric gp130 co-receptors. In the *trans*-signaling pathway, secreted IL-6 binds to the soluble form of IL-6R (sIL-6R) to further transmit the signal via binding to the gp130 co-receptor on the membrane surface. Thus, the *trans*-signaling allows stimulation of cells that do not express membrane-bound IL-6R but the gp130 co-receptor^15^. Based on the finding that IL-6 promotes osteoclast formation only in the presence of sIL-6R^10,16^, IL-6 seems to stimulate osteoclastogenesis via the *trans*-signaling pathway. On the other hand, osteoclast precursors and mature osteoclasts express both IL-6R and gp130^17–20^, suggesting that osteoclasts may have the capacity to respond to IL-6 via the classic signaling pathway as well. In fact, studies in human osteoclast cultures support this idea^8,9,21^. In addition, it has been proposed that IL-6 modulates osteoclastogenesis induced by RANKL-producing osteoblasts^16,22,23^. Thus, it remains controversial which target cells are stimulated by IL-6 and which role IL-6 plays in basal and pathological bone resorption.

Besides the membrane-bound gp130, a soluble form of gp130 (sgp130) functions as a natural inhibitor of IL-6 *trans*-signaling as it competes with the membrane-bound gp130 for the formation of the complex with IL-6/sIL-6R^14^. Since sgp130 binds IL-6 only in the presence of sIL-6R, it specifically inhibits the *trans*-signaling, while the classic signaling pathway remains unaffected^24^. Therefore, sgp130 represents a blocking agent to neutralize circulating IL-6^25^ and provides a basis for the treatment of osteoporosis and other skeletal diseases associated with elevated IL-6 levels.

Severe skeletal pathologies and growth retardation are typical symptoms in patients with mucolipidosis type II (MLII), a rare lysosomal storage disorder of childhood^26^. MLII is caused by mutations in the *GNPTAB* gene encoding the catalytic α- and β-subunits of the GlcNAc-1-phosphotransferase, which generates mannose 6-phosphate residues on lysosomal enzymes for their efficient delivery to lysosomes^27–29^. In cells from patients with MLII, missorting and hypersecretion of lysosomal enzymes lacking the mannose 6-phosphate targeting signals results in accumulation of non-degraded material in lysosomes and subsequently impairs the function of various cell types and tissues^28^. The systematic skeletal analysis of MLII mice, harboring an MLII patient mutation, revealed that the progressive bone loss observed in these animals is caused by dysfunction of bone-forming osteoblasts and increased number of bone-resorbing osteoclasts^30^. Importantly, since the expression of IL-6 was strongly increased in primary cultured MLII osteoblasts^29^, we hypothesized that the osteoporotic phenotype in MLII is mainly caused by excessive osteoclastogenesis, which is potentially induced by osteoblast-derived IL-6.

In this study we have focused on a potential treatment option to prevent the bone loss and skeletal deformities in MLII mice. More specifically, we addressed the clinically relevant question whether a blockade of IL-6 *trans*-signaling can reduce the excessive osteoclastogenesis in MLII mice and, subsequently normalize their bone remodeling pathology.

## Results

### Increased expression of IL-6 in terminally differentiated primary osteoblasts and chondrocytes from MLII mice

**Figure 1:**
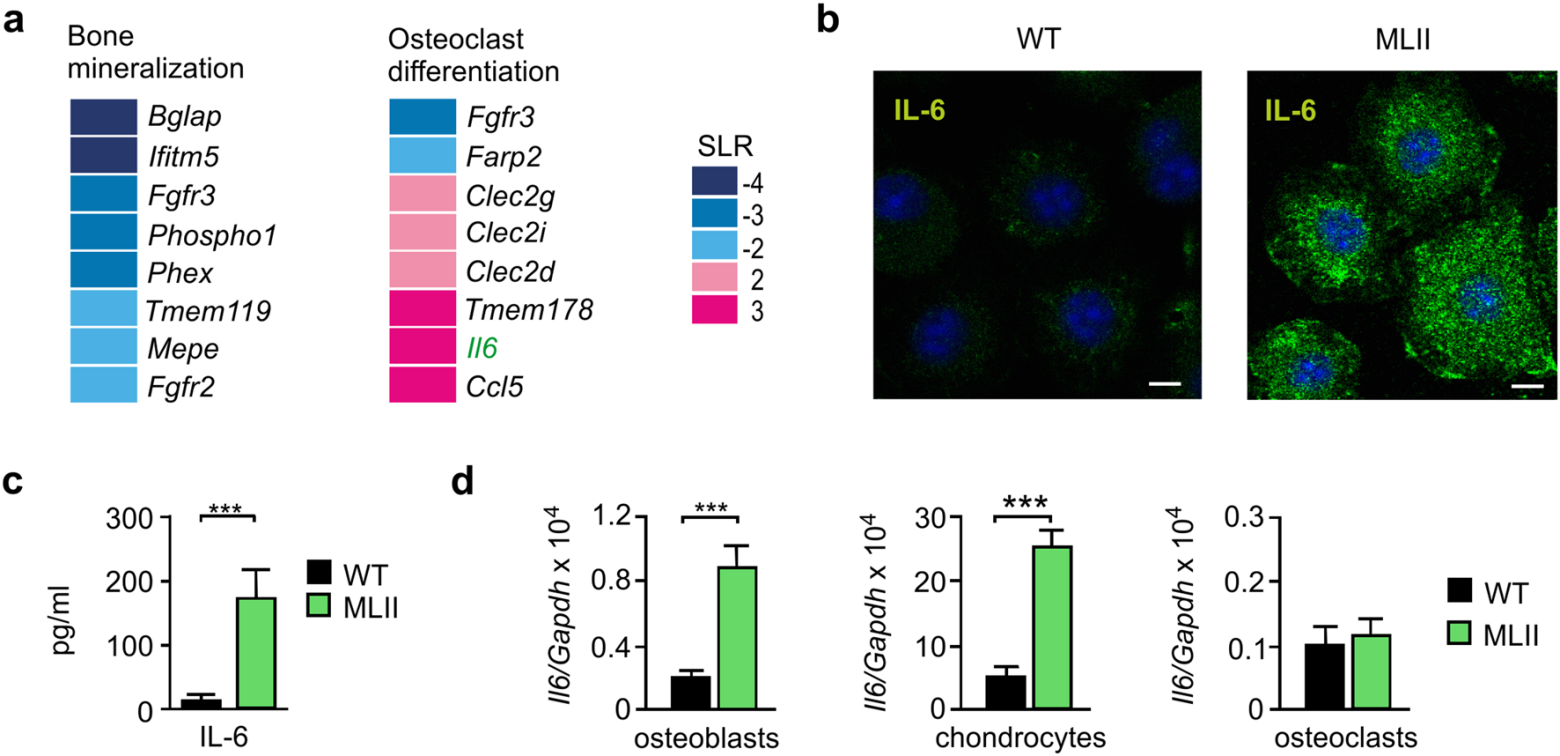
Increased expression of IL-6 in primary cultured osteoblasts and chondrocytes from MLII mice. (**a**) Signal log ratio (SLR) of differentially expressed genes (SLR ≥ 2 or ≤ -2) in terminally differentiated osteoblasts from wild-type (WT) and MLII mice related to the gene ontology (GO) "Bone biological processes": "Bone mineralization" (GO 0030282) and "Osteoclast differentiation" (GO 0030316). (**b**) IL-6 immunostaining (green) of WT and MLII osteoblasts. Nuclei were visualized by 4′,6-diamidino-2-phenylindole (DAPI) staining (blue). Scale bar: 10 μm. (**c**) Concentration of IL-6 in conditioned media from WT and MLII osteoblasts (n = 5, mean ± SD, ***p ≤ 0.001). (**d**) Expression levels of *Il6* mRNA related to *Gapdh* in primary osteoblasts, chondrocytes and osteoclasts from WT and MLII mice (n = 3, mean ± SD, ***p ≤ 0.001).

Osteoblast differentiation requires the coordinated stepwise expression of multiple genes. Previously we have shown that most of these genes are expressed at lower levels in MLII osteoblasts during the early stage of osteoblastogenic differentiation^30^. Since lysosomal storage accumulation was more pronounced in mature MLII osteoblasts^30^, we aimed to assess genome-wide transcriptional differences between wild-type and MLII osteoblasts, which were cultured for 25 days to reach an osteocyte-like state of terminal differentiation^31^. The transcriptome and gene ontology (GO) enrichment analysis revealed that dysregulated genes related to the GO-category "Bone biological processes" were significantly enriched in MLII osteoblasts. The increased or decreased mRNA expression pattern was associated with the GO-terms "Bone mineralization" or "Osteoclast differentiation" (Fig. 1a, Supplementary Material, Table S1). More specifically, the transcription of genes important for bone mineralization (e.g. *Bglap, Ifitm5, Phex* and *Mepe*) was strongly decreased in MLII osteoblasts, which was confirmed by quantitative PCR analysis (Supplementary Material, Fig. S1). On the other hand, osteoclastogenic factors such as the cytokines *Il6* and *Ccl5*^16,32^ were significantly induced in osteoblasts from MLII mice (Fig. 1a, Supplementary Material, Fig. S1).

Next, we confirmed the elevated expression of *Il6* transcripts in MLII osteoblasts on the protein level. For this purpose, we performed IL-6 immunostaining of primary wild-type and MLII osteoblasts, which revealed strongly increased levels of intracellular IL-6 in MLII cultures (Fig. 1b). Besides, MLII osteoblasts excessively secreted IL-6 into the cell culture medium (Fig. 1c). A fivefold increased *Il6* transcript level was also found in primary chondrocytes of MLII mice, whereas the *Il6* mRNA expression in primary MLII osteoclasts was not affected (Fig. 1d). Interestingly, we found the basal mRNA level of *Il6* to be very low in wild-type osteoblasts and osteoclasts, whereas in chondrocytes of the cartilage the *Il6* transcript concentration was approximately 50-fold higher than in bone cells (Fig. 1d).

These *in-vitro* studies corroborated our previous findings in osteoblasts at the early stage of differentiation^30^ and suggested that the progressive bone loss and osteoporotic phenotype in MLII might be caused by osteoblast dysfunction and an increased expression of the osteoclastogenic factor IL-6 derived from MLII osteoblasts and chondrocytes.

### In-vitro differentiation of primary osteoclasts is enhanced via IL-6 classic and *trans*-signaling

**Figure 2:**
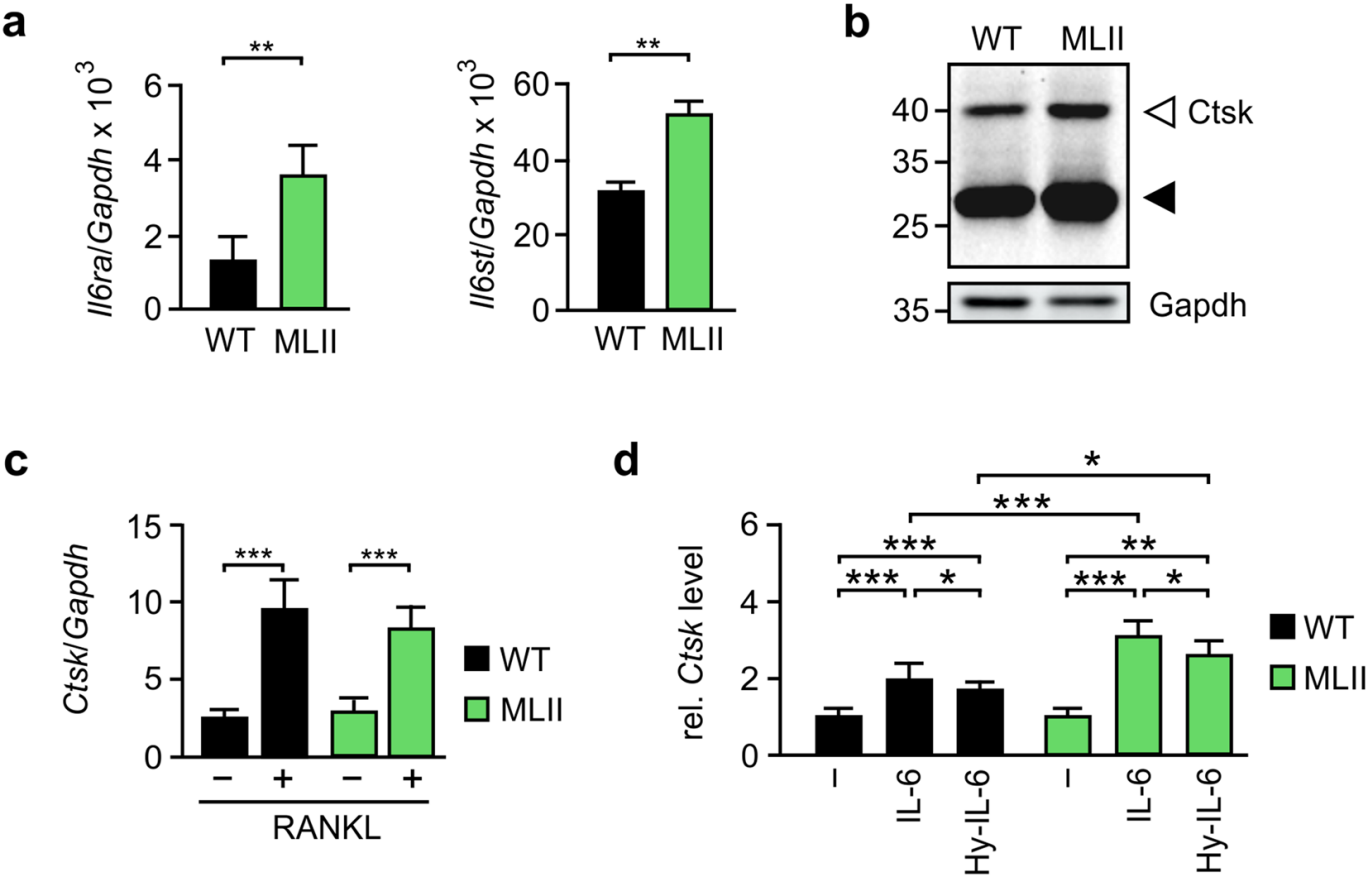
*In-vitro* osteoclastogenesis can be mediated by IL-6 classic and *trans*-signaling. (**a**) mRNA Expression levels of *Il6ra* (encoding IL-6R) and *Il6st* (encoding gp130) related to *Gapdh* in primary osteoclasts from wild-type (WT) and MLII mice (n = 3, mean ± SD, **p ≤ 0.01). (**b**) Representative western blot analysis of extracts (25 µg protein) of WT and MLII osteoclasts using an antibody against cathepsin K (Ctsk). The positions of precursor (open arrowhead) and mature form (black arrowhead) are indicated. Endogenous Gapdh was used as loading control. (**c**) mRNA expression levels of *Ctsk* related to *Gapdh* in primary osteoclasts from WT and MLII mice cultured in the presence or absence of 40 ng/ml RANKL (n = 3, mean ± SD, ***p ≤ 0.001). (**d**) Relative mRNA expression levels of *Ctsk* related to *Gapdh* in primary osteoclast cultures from WT and MLII mice (supplemented with 40 ng/ml RANKL) in the presence or absence of 100 ng/ml IL-6 and/or 100 ng/ml hyper-IL-6 (Hy-IL-6) as indicated (n = 3, mean ± SD, *p ≤ 0.05, **p ≤ 0.005, ***p ≤ 0.001).

To study the responsiveness of primary osteoclasts from wild-type and MLII mice to IL-6 we first determined the mRNA levels of *Il6ra* (encoding IL-6R) and *Il6st* (encoding gp130) by quantitative PCR in bone marrow cells incubated for 7 days in the presence of the osteoclastogenic factors 1,25-dihydroxyvitamin-D3, M-CSF and RANKL. According to previous findings^17–20^, both receptors were found to be expressed in wild-type osteoclasts, with the mRNA level of *Il6st* being 30-fold higher compared to *Il6ra* (Fig. 2a). Interestingly, transcription of both genes was significantly increased in MLII osteoclasts (Fig. 2a).

The formation of multinucleated giant cells is an indicator for proper osteoclast differentiation. These multinucleated cells (MNC) were visualized by staining with the osteoclast marker protein TRAP (lysosomal tartrate-resistant acid phosphatase) and were mainly detectable in RANKL-supplemented cultures (Supplementary Material, Fig. S2a). When we differentiated wild-type bone marrow cells in the presence of recombinant IL-6, a significant increase in the RANKL-dependent osteoclastic MNC formation was observed (Supplementary Material, Fig. S2b). Due to lacking mannose 6-phosphate formation on lysosomal enzymes in MLII cells, TRAP is less present intracellularly^30^ (Supplementary Material, Fig. S2c). The osteoclast marker *Ctsk* (encoding lysosomal cathepsin K) is also transcriptionally activated during osteoclast differentiation of bone marrow cells^33^. However, unlike TRAP, the newly synthesized Ctsk was not mistargeted to the extracellular space in MLII cells, and its intracellular amounts were comparable to those in wild-type cells, which were demonstrated by the presence of the lysosomal mature forms of the enzyme in both cell types (Fig. 2b). Therefore, we quantified and compared the osteoclast formation of wild-type and MLII cells in response to IL-6 by measuring the mRNA expression of *Ctsk* instead of TRAP activity staining. As a proof of principle, we thereby confirmed the previous finding^30^ that the RANKL-dependent differentiation of MLII osteoclasts was comparable to wild-type cultures (Fig. 2c). In addition, the osteoclast formation was enhanced in wild-type and MLII cultures in the presence of IL-6, which is known to induce osteoclastogenesis via both classic and *trans*-signaling (Fig. 2d). A similar but less pronounced effect was observed in the presence of hyper-IL-6 (Fig. 2d), an activator of *trans*-signaling which represents a recombinant IL-6 fused to the sIL-6R via a flexible peptide linker^34,35^. Importantly, both signaling pathways were found to be significantly enhanced ~ 1.6-fold in MLII osteoclasts differentiated in the presence of either IL-6 or hyper-IL-6, as compared to the respectively treated wild-type cultures (Fig. 2d). This was most likely caused by the elevated expression of IL-6R and gp130 in MLII cells (Fig. 2a). Similar to wild-type osteoclasts, hyper-IL-6 enhanced osteoclastogenesis in MLII cultures less strongly than IL-6, suggesting the involvement of both classic and *trans*-signaling in osteoclast formation (Fig. 2d).

### Reduced bone mass and increased osteoclastogenesis in MLII mice is not prevented by IL-6 *trans*-signaling inhibition

The obtained *in-vitro* results led us to further expand our analysis by addressing the potential relevance of IL-6 in activated osteoclastogenesis of MLII mice *in-vivo*. Thereby we aimed to inhibit the increased local bone-specific IL-6 production and to prevent bone loss in MLII. Since IL-6 mediates the activation of anti-inflammatory pathways in the immune system^14^, the complete block of IL-6 signaling might lead to serious side effects. Thus, we decided rather to reduce the potency of IL-6 in MLII mice by blocking specifically the IL-6 *trans*-signaling pathway by the use of soluble gp130Fc. Since the early development and prenatal or neonatal onset of MLII-associated skeletal alterations in humans and mice^26,30^, we decided for a transgenic approach for constant sgp130Fc expression in the circulation from the birth instead of sgp130Fc protein injection.

**Figure 3:**
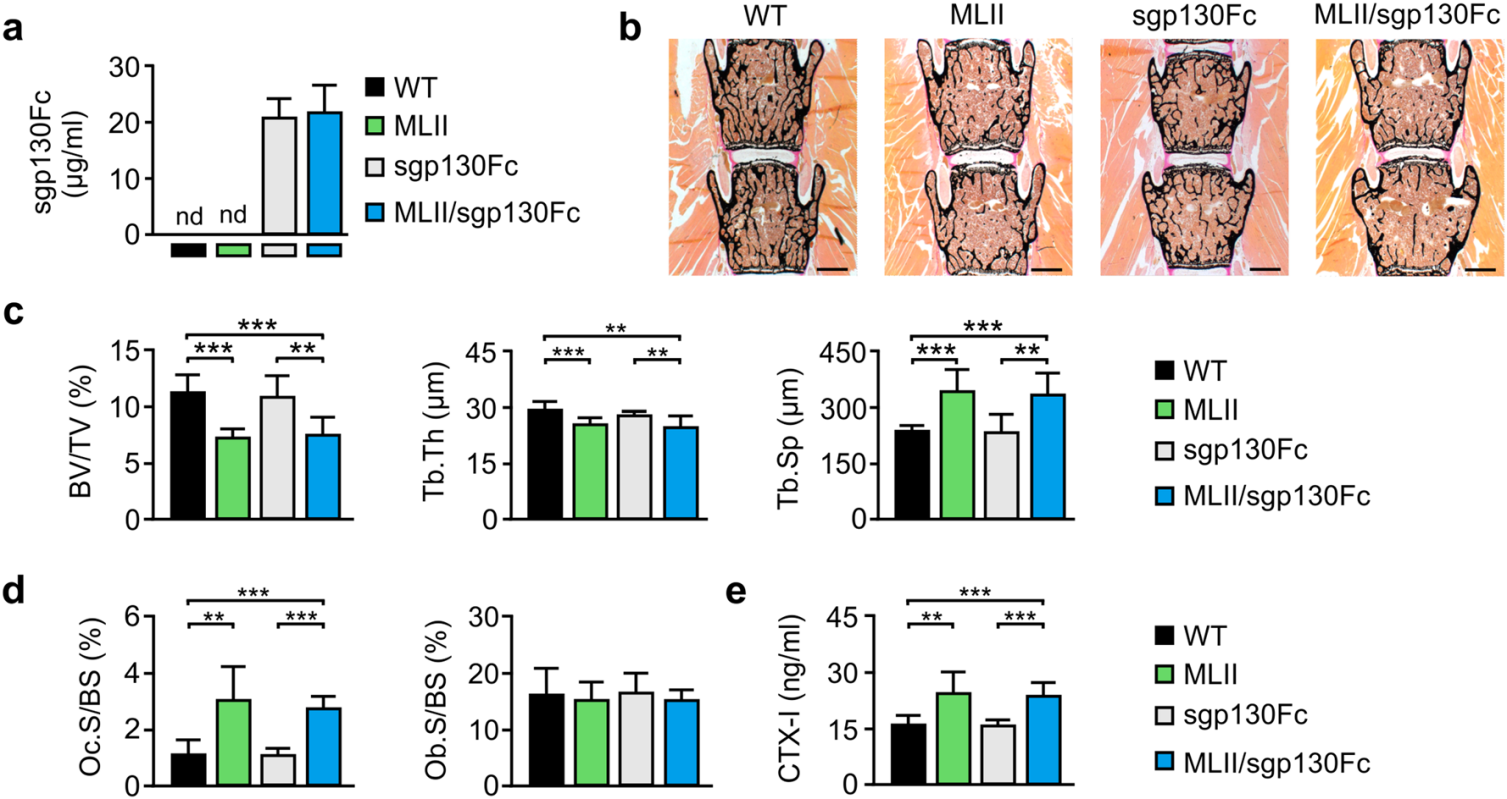
Reduced trabecular bone mass of female 12-week-old MLII mice is not prevented by sgp130Fc. (**a**) Concentration of sgp130Fc protein in serum from wild-type (WT), MLII, sgp130Fc and MLII/sgp130Fc mice (n ≥ 5, mean ± SD). nd: not detected. (**b**) Representative von Kossa/van Gieson staining of undecalcified vertebra sections from WT, MLII, sgp130Fc and MLII/sgp130Fc mice (scale bars: 1 mm). (**c**) Quantification of the vertebral trabecular bone volume per tissue volume (BV/TV), trabecular thickness (Tb.Th) and trabecular spacing (Tb.Sp) from the same mice (n = 5, mean ± SD, **p ≤ 0.005, ***p ≤ 0.001). (**d**) Quantification of cellular parameters in vertebra sections from the same mice: osteoclast surface per bone surface (Oc.S/BS) and osteoblast surface per bone surface (Ob.S/BS) (n = 5, mean ± SD, **p ≤ 0.005, ***p ≤ 0.001). (**e**) Concentration of C-terminal telopeptides of type I collagen (CTX-I) in serum from WT, sgp130Fc, MLII and MLII/sgp130Fc mice (n ≥ 7, mean ± SD, **p ≤ 0.005, ***p ≤ 0.001).

Therefore we crossed heterozygous MLII mice with transgenic mice expressing the human recombinant fusion protein sgp130Fc, which comprises the soluble extracellular portion of gp130 with the constant portion of the mouse IgG1 antibody^36^. The expression is driven by the liver-specific PEPCK promoter and results in the concentration of sgp130Fc as high as 20–30 µg/ml in the circulation of transgenic mice^36^. Accordingly, we detected approximately 25 µg/ml sgp130Fc in the serum of sgp130Fc and MLII/sgp130Fc, which was not present in wild-type and MLII mice, as expected (Fig. 3a). For skeletal analysis, we performed histology and quantitative histomorphometry of undecalcified vertebra sections from 12-week-old wild-type, MLII, sgp130Fc and MLII/sgp130Fc mice (Fig. 3b–d). Similar to our previous data^30^, we found by von Kossa/van Gieson staining of the mineralized bone matrix that the trabecular bone volume was significantly reduced in MLII mice, which was also reflected by decreased trabecular thickness and increased trabecular spacing (Fig. 3b,c). However, we did not observe significant differences in the trabecular bone parameters between MLII and MLII/sgp130Fc mice. Furthermore, we confirmed that the low trabecular bone mass in MLII mice results from a highly elevated number of osteoclasts, while the osteoblast number was not affected in 12-week-old MLII mice (Fig. 3d). Likewise, the quantification of the bone resorption biomarker CTX-I in the serum revealed that MLII mice display excessive bone resorption (Fig. 3e). Again, we did not observe significant differences between MLII and MLII/sgp130Fc mice, since excessive bone resorption was also detected in the latter. These analyses demonstrated that the blockade of the IL-6 *trans*-signaling pathway by sgp130Fc does not normalize increased osteoclast differentiation to prevent the osteoporotic phenotype in MLII mice. Furthermore, and in contrast to a previous study reporting that high doses of sgp130Fc result in impaired growth and low bone mass in mice^37^, we did not observe any detrimental effect of sgp130Fc on the skeleton.

### IL-6 *trans*-signaling inhibition does not prevent growth retardation in MLII mice

Skeletal growth is primarily dependent on the coordinated differentiation of growth plate chondrocytes^38^. MLII mice at the age of 4 and 12 weeks are characterized by growth retardation accompanied by growth plate widening^30^. Similarly, transgenic IL-6 mice are also reduced in size and display defective growth plates^39,40^. Interestingly, *Il6* mRNA was found to be much higher expressed in chondrocytes compared to bone cells (Fig. 1d). These findings indicate an important role of IL-6 in skeletal development.

**Figure 4:**
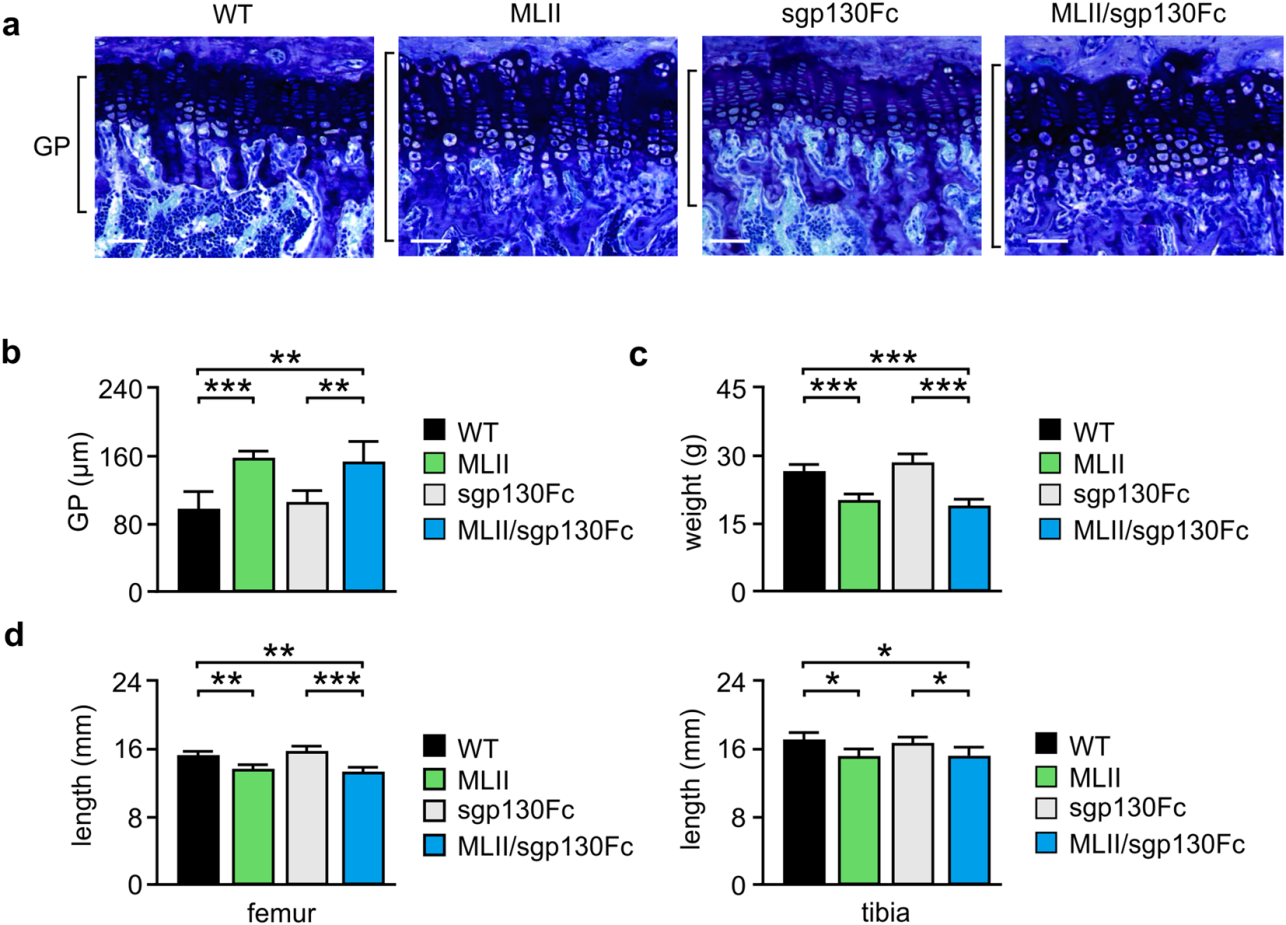
Retarded growth of female 12-week-old MLII mice is not prevented by sgp130Fc. (**a**) Representative toluidine blue staining of the growth plate in undecalcified tibia sections from wild-type (WT), MLII, sgp130Fc and MLII/sgp130Fc mice. Scale bars: 1 mm. (**b**) Quantification of the growth plate width in tibia of the same mice (n ≥ 4, mean ± SD, **p ≤ 0.005, ***p ≤ 0.001). (**c**) Body weight of WT, MLII, sgp130Fc and MLII/sgp130Fc mice (n ≥ 7, mean ± SD, ***p ≤ 0.001). (**d**) Femur and tibia length of WT, MLII, sgp130Fc and MLII/sgp130Fc mice (n ≥ 5, mean ± SD, *p ≤ 0.05, **p ≤ 0.005, ***p ≤ 0.001).

We therefore performed toluidine-blue staining of the growth plates in undecalcified tibia sections from 12-week-old mice and found that the growth plate width was significantly increased in MLII and MLII/sgp130 mice compared to wild-type and sgp130Fc mice (Fig. 4a,b). This was accompanied by reduced body weight as well as decreased femur and tibia length of MLII and MLII/sgp130 mice in comparison to the respective controls (Fig. 4c,d). Similar results were obtained in 4- and 50-week-old animals (Supplementary Material, Fig. S3a). Of note, the dramatic body weight loss in old MLII and MLII/sgp130 mice is additionally enhanced by the general pathological constitution including severe neurodegeneration in MLII mice^41^. In addition, similar to 12-week-old mice, the severe bone remodeling phenotype of 50-week-old MLII mice was not corrected by sgp130Fc (Supplementary Material, Fig. S3b). Taken together, transgenic inhibition of IL-6 *trans*-signaling could not normalize the skeletal abnormalities of MLII mice.

## Discussion

Osteoporosis, representing the most common bone disorder and one of the most prevalent diseases in the aged population, is primarily caused by increased bone resorption mediated by osteoclasts^42^. It is therefore of high clinical relevance that there is accumulating evidence indicating that increased IL-6 production in pathological conditions, such as inflammation, induces osteoclastogenesis^23^. More specifically, enhanced IL-6 production has been reported to be associated with bone loss in postmenopausal women^12^. Similarly, despite showing no skeletal abnormalities in the absence of specific challenges, IL-6^-/-^ mice were reported to be protected from bone loss induced by ovariectomy^43^. Likewise, IL-6 transgenic mice with high circulating IL-6 levels are characterized by an increased number of osteoclasts, causing bone loss and alterations of the skeletal microstructure^40^. Furthermore, bone loss induced by spaceflight was found to be associated with increased IL-6 expression in rodents^44,45^. Importantly however, the precise role of IL-6 in physiological bone remodeling and skeletal maintenance is not fully understood so far, especially regarding the responsible signaling pathways.

MLII is a severe multi-systemic disease resulting in premature death during early childhood. To date, there is no established treatment to cure MLII. Although the skeletal alterations associated with MLII*,* such as short stature, bowed limbs, progressive joint stiffness, hip and knee contractures, thoracal asymmetry and kyphoscoliosis, do not contribute to premature mortality, they lead to chronic pain, progressive decline of mobility and social stigmatization of the affected children^26^. Since patients with MLII are also characterized by progressive bone loss, it was important that our previous analysis of a corresponding mouse model revealed that these animals display a remarkable increase of osteoclastogenesis. Consistently, the osteoporotic phenotype of MLII mice was prevented by administration of the established anti-resorptive bisphosphonate alendronate^30^. However, as long-term bisphosphonate administration, due to its interference with physiological bone remodeling^42,46^, is not a recommended treatment for children, it is relevant to establish alternative therapeutic options to prevent bone loss and skeletal deformities in MLII.

Our previous analyses of MLII mice clearly demonstrated that these animals recapitulate the hallmark clinical symptoms observed in MLII patients, thereby underscoring their value as a disease model. Importantly, the skeletal phenotype of MLII mice, i.e. low bone mass due to increased osteoclastogenesis, was associated with increased local expression of IL-6 in the bone microenvironment. In the present manuscript we studied the relevance of this previous observation to address two major questions. Is the unexpected induction of IL-6 expression in skeletal cell types of MLII mice causing their osteoporotic phenotype? Is blockade of IL-6 *trans*-signaling a potential therapeutic option to prevent bone loss in MLII?

Our present study demonstrated that IL-6 is released from osteoblasts to stimulate RANKL-dependent osteoclast formation. Noteworthy, the osteoclastogenesis appeared to be mediated via IL-6 classic and *trans*-signaling, both pathways being enhanced in osteoclasts from MLII mice. This observation prompted us to reduce the potency of IL-6 in MLII mice via blocking selectively the IL-6 *trans*-signaling pathway by recombinant sgp130Fc, which represents a novel therapeutic agent for the treatment of chronic inflammatory diseases^47^. Importantly, since spg130Fc has been suggested to prevent side effects triggered by a global IL-6 signaling blockade, it is principally an available treatment option for patients with MLII. However, deep skeletal phenotyping of MLII and MLII/sgp130Fc mice revealed that bone loss in MLII mice cannot be prevented by blockade of IL-6 *trans*-signaling with sgp130Fc. It is also important to state that we did not observe an impact of high circulating sgp130Fc levels on bone mass and skeletal remodeling on a wild-type genetic background. Thus, although IL-6 signaling represents a potential anti-resorptive therapeutic target, the specific role of classic and trans-IL-6 signaling is highly demanded to treat bone diseases associated with elevated IL-6 levels.

## Methods

### Mice

Generation and genotyping of MLII mice and sgp130Fc transgenic mice was described elsewhere^36,41^. Heterozygous MLII mice were crossed with sgp130Fc transgenic mice to generate homozygous MLII-sgp130Fc animals. Wild-type (WT), sgp130Fc and MLII littermates were used as controls. All mice were kept in a pathogen-free environment with a 12-h light/dark cycle, 45% to 65% relative humidity and 20 °C to 24 °C ambient temperature in open or individually ventilated cages with wood shavings bedding and nesting material in groups not surpassing 6 animals. The mice had access to tap water and standard rodent chow ad libitum. All experimental procedures were performed according to the institutional guidelines and approved by the “Behörde für Gesundheit und Verbraucherschutz”. The study was carried out in compliance with the ARRIVE guidelines (http://www.nc3rs.org.uk/page.asp?id=1357).

### Primary osteoblasts

For transcriptome analysis, osteoblast progenitors were isolated from individual calvariae of 8 to 10 mice at the age of 3 to 5 days. Cells were released by collagenase/dispase digestion and plated in α-MEM containing 10% FBS (α-MEM/FBS) at an initial density of 10,000 cells per cm^2^. Osteoblast differentiation was induced at 80% confluency by the addition of 50 µg/ml ascorbic acid and 10 mM β-glycerophosphate (both from Sigma-Aldrich) followed by culture for 25 days to induce an osteocyte-like state of terminal differentiation.

For all other experiments, bone marrow was flushed out of the femora from 6 to 8 mice at the age of 12 weeks with α-MEM/FBS. Cells were then plated at a density of 5 × 10^6^ cells per ml, and after 24 h the adherent cells were cultured for 25 days in α-MEM/FBS containing 50 µg/ml ascorbic acid and 10 mM β-glycerophosphate.

### Primary osteoclasts

For osteoclast differentiation, bone marrow was flushed out of the femora from 6 to 8 mice at the age of 12 weeks with α-MEM/FBS. Cells were then plated at a density of 5 × 10^6^ cells per ml, and after 24 h the adherent cells were cultured in α-MEM/FBS containing 10 nM 1,25-dihydroxyvitamin-D3 (Sigma-Aldrich). Beginning at day 4 after seeding M-CSF and RANKL (both from Peprotech) were added to a final concentration of 20 ng/ml and 40 ng/ml, respectively, and the cells were cultured for 7 days to generate osteoclasts. For the stimulation experiments, 100 ng/ml IL-6^47^ and 100 ng/ml hyper-IL-6^34,48^ were added to the culture medium during the whole period of osteoclast differentiation.

Formation of multinuclear cells was assessed by tartrate-resistant acid phosphatase (TRAP) activity staining as described previously^49^. In brief, after removal of the medium and two washing steps with phosphate-buffered saline (PBS), cells were fixed with cold methanol for 5 min. After washing and drying, cells were stained with Naphthol AS-MX-Phosphate (Sigma-Aldrich) for 30 min before the number of TRAP-positive multinuclear cells per well was counted.

### Primary chondrocytes

Chondrocyte progenitor cells were isolated from a single sternum of 8 to 10 wild-type and *Gnptg*^*ko*^ mice at the age of 10 days. Cells were separated by digesting the tissue initially in 0.1% collagenase Ia solution followed by 0.2% collagenase Ia solution and cultured in DMEM/Ham’s F‐12 (1:1) medium supplemented with 10% FCS. At a total cell confluence of 80%, chondrocyte differentiation was induced by the addition of ascorbic acid (50 µg/ml) and culture for 10 days.

### Transcriptome analysis

Total RNAs from calvarial osteoblasts of each four WT and MLII mice were isolated with the PEQ Gold Total RNA Isolation Kit (VWR) according to manufacturer's instructions. The genome-wide gene expression analysis on ArrayXS Agilent microarrays was performed by OakLabs (Henningsdorf, Germany) using the mouse GE 8 × 60 k v2 Affymetrix microarray. Quality control was performed using arrayQualityMetrics and quantile normalization of the expression data was performed with limma^50,51^. Probes for the same gene were summarized to the median intensity. Surrogate variable analysis was performed to correct for batch effects and the resulting co-variants were incorporated into limma to identify differentially expressed genes^52^. Benjamini–Hochberg correction was performed to correct for multiple testing. For gene ontology enrichment analysis, differentially expressed genes (absolute 1.5 SLR and adjusted *p* value ≤ 0.05) were used to find enriched gene ontology biological processes using GOseq^53^. Gene ontology terms with an adjusted *p* value ≤ 0.05 were regarded as statistically significant.

For quantitative mRNA expression analysis, RNA isolation from cultured cells, cDNA synthesis and quantitative PCR using pre-designed Taqman-Assays (Thermo Fisher Scientific) were performed as previously described^54^. The relative mRNA expression levels of analyzed genes were normalized to the level of *Gapdh* mRNA in the same cDNA using the comparative CT method (2^–ΔΔCT^).

### Protein analysis

For western blot analysis, cells were lysed in PBS containing 0.5% Triton X-100 and protease inhibitors for 30 min at 4 °C. After centrifugation at 16,000*g* supernatants were used for measurement of the protein content. Cell extracts (25 µg protein) were processed for SDS-PAGE and western blot analysis using the mouse anti-Ctsk or rabbit anti-Gapdh antibodies (both from Santa Cruz Biotechnology), the latter serving as loading control, as previously described^54^.

Concentration of IL-6 in cell culture supernatants of primary osteoblasts was quantified by mouse IL-6 ELISA (KMC0061; Thermo Fisher Scientific) according to the manufacturers' instructions. The activity of TRAP in cell extracts was determined as described previously^55^. Serum concentration of bone-specific collagen degradation products (C-terminal telopeptides of type I collagen, CTX-I) was determined by ELISA (Immunodiagnostic Systems). Serum concentrations of human sg130Fc protein in 4- and 12-week-old transgenic mice was determined by the DuoSet human gp130 ELISA Kit (R&D Systems) according to the manufacturers' instructions.

### Immunofluorescence microscopy

Primary cultured osteoblasts grown on cover slips were fixed with 4% paraformaldehyde in PBS for 30 min. After washing with 50 mM ammonium chloride, cells were permeabilized with 0.1% saponine in PBS for 10 min and blocked in PBS containing 0.1% saponine and 3% bovine serum albumin for 30 min. Subsequently, cells were incubated with rabbit anti-IL-6 antibody (MAB406; R&D Systems) for 2 h. After washing with 0.1% saponine in PBS, cells were incubated with corresponding secondary antibodies conjugated to Alexa Fluor 488 (Thermo Fisher Scientific) and 4′,6-diamidino-2-phenylindole (DAPI) for 1 h and embedded in Aqua-Poly/Mount. Fluorescence was detected and images were obtained using an Olympus digital scanning confocal microscope (FluoView F1000) and Adobe Photoshop and Image J software.

### Skeletal analysis

Dissected skeletons were fixed in 3.7% PBS-buffered formaldehyde for 18 h at 4 °C and stored in 80% ethanol. All skeletons were first analyzed by contact radiography (Faxitron Xray) to measure the length of femora and tibia. For non-decalcified bone histology, the lumbar vertebral bodies L1 to L4 of each mouse were dehydrated in ascending alcohol concentrations and then embedded in methylmetacrylate as described previously^49^. Sections of 4 μm thickness were cut sagittally on a Microtec rotation microtome (Techno-Med GmbH) and stained by von Kossa/van Gieson procedures. Histomorphometry was performed according to the ASBMR guidelines^56^ using the OsteoMeasure system (Osteometrics).

### Statistical analysis

Results were expressed as means ± standard deviations (SD) and the values were compared using the unpaired Student’s *t*-test. Value differences with *p* < 0.05, *p* < 0.005 and *p* values < 0.001 were considered statistically significant, whereas *, ** or *** indicate the significance levels.

## Supplementary Information

Supplementary Information.
