# Supplementary material for: Transgenic inhibition of interleukin-6 trans-signaling does not prevent skeletal pathologies in mucolipidosis type II mice
Source: Sci Rep. 2021 Feb 11;11:3556. doi: 10.1038/s41598-021-82802-3 (PMC7878873; doi:10.1038/s41598-021-82802-3)
Supplement: Supplementary file 1 — Supplementary Information. [file 41598_2021_82802_MOESM1_ESM.pdf]

Supplementary Tables and Figures

Transgenic inhibition of interleukin-6 *trans*-signaling does not prevent skeletal pathologies in mucolipidosis type II mice

Lena Marie Westermann, Anke Baranowsky, Giorgia Di Lorenzo, Tatyana Danyukova, Jamie Soul, Jean-Marc Schwartz, Gretl Hendrickx, Michael Amling, Stefan Rose-John, Christoph Garbers, Thorsten Schinke, Sandra Pohl

Supplementary Table S1. Mean signal and signal log ratio (SLR) of differentially expressed genes in terminally differentiated osteoblasts from MLII mice compared to wild-type (WT) controls related to gene ontology (GO) biological processes: "Bone mineralization" (GO: 0030282) and "Osteoclast differentiation" (GO: 0030316).

**Gene**	**Name**	**Mean signal**		**SLR**	**P-Value**
		**WT**	**MLII**	**Log2FC**	**(< 0.05)**
**Bone mineralization**					
Bglap	*bone gamma-carboxyglutamate protein*	*71574*	*4567*	*-4.0*	*0.0001*
Ifitm5	*interferon induced transmembrane protein 5*	*10961*	*699*	*-4.0*	*0.0001*
Fgfr3	*fibroblast growth factor receptor 3*	*6167*	*912*	*-2.8*	*0.0008*
Phospho1	*phosphatase, orphan 1*	*21353*	*3383*	*-2.6*	*0.0001*
Phex	*phosphate regulating endopeptidase homolog, X-linked*	*2488*	*456*	*-2.6*	*0.0038*
Tmem119	*transmembrane protein 119*	*47183*	*9450*	*-2.4*	*0.0034*
Mepe	*matrix extracellular phosphoglycoprotein*	*446*	*101*	*-2.2*	*0.0036*
Fgfr2	*fibroblast growth factor receptor 2*	*18176*	*4250*	*-2.1*	*0.0003*
**Osteoclast differentiation**					
Fgfr3	fibroblast growth factor receptor 3	6167	912	-2.8	0.0008
Farp2	FERM, RhoGEF and pleckstrin domain protein 2	2574	574	-2.2	0.0001
Clec2g	C-type lectin domain family 2, member g	60	219	1.9	0.0013
Clec2i	C-type lectin domain family 2, member i	88	347	2.0	0.0002
Clec2d	C-type lectin domain family 2, member d	229	910	2.0	0.0033
Tmem178	transmembrane protein 178	285	1963	2.6	0.0059
Il6	interleukin-6	4	31	3.1	0.0001
Ccl5	chemokine (C-C motif) ligand 5	33	399	3.6	0.0002

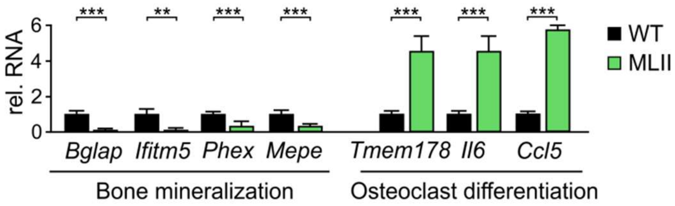

Supplementary Figure S1. Relative mRNA expression levels of indicated genes linked to the GO-terms "Bone mineralization" and "Osteoclast differentiation" in wild-type (WT) and MLII osteoblasts (n = 3, mean ± SD, **$P \leq 0.005$, ***$P \leq 0.001$).

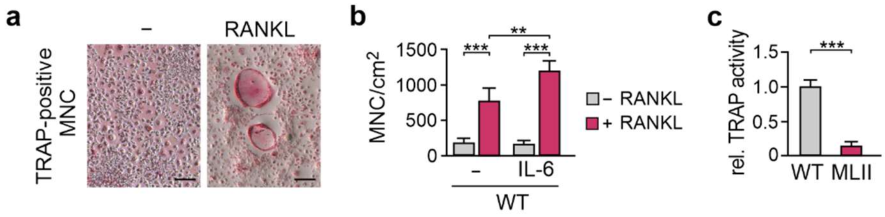

Supplementary Figure S2. *In-vitro* osteoclastogenesis can be mediated by IL-6 classic and *trans*-signaling. (**a**) Representative tartrate-resistant acid phosphatase (TRAP) activity staining of wild-type multinucleated cells (MNC) in the presence or absence of 40 ng/ml RANKL. Scale bars = 50 µm. (**b**) Quantification of wild-type (WT) TRAP-positive MNC in the presence or absence of 40 ng/ml RANKL and 100 ng/ml IL-6 as indicated (n = 3, mean ± SD, *P ≤ 0.05, **$P \leq 0.005$***, $P \leq$ 0.001). (**c**) Relative enzyme activity of TRAP in extracts of WT and MLII osteoclasts (n = 6, mean ± SD, ***$P \leq 0.005$).

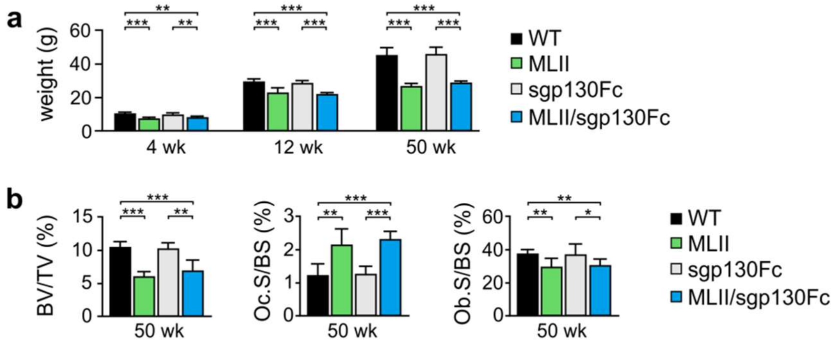

Supplementary Figure S3. Growth retardation and reduced trabecular bone mass of male MLII mice is not prevented by sgp130Fc. (**a**) Body weight of 4- 12- and 50-week-old wild-type (WT), MLII, sgp130Fc and MLII/sgp130Fc mice (n ≥ 5, mean ± SD, **$P \leq 0.005$, ***$P \leq 0.001$). (**b**) Quantification of the vertebral trabecular bone volume per tissue volume (BV/TV), osteoclast surface per bone surface (Oc.S/BS) and osteoblast surface per bone surface (Ob.S/BS) of 50-week-old WT, MLII, sgp130Fc and MLII/sgp130Fc mice (n = 5, mean ± SD, *$P \leq 0.05$, **$P \leq 0.005$, ***$P \leq$ 0.001).

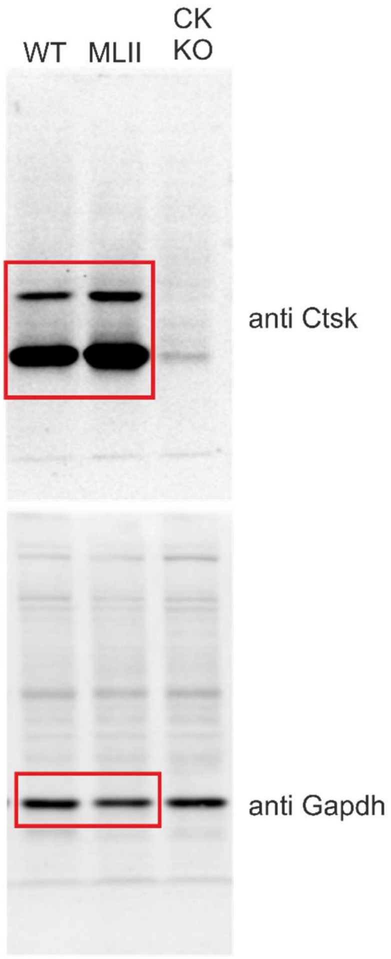

Supplementary Figure S4. Original, non-cropped western blots related to Fig. 2b.
